# Investigation of Degradation Behavior and Mechanical Performance Deterioration of Magnesium Alloys in Hank’s Solution

**DOI:** 10.3390/ma18225102

**Published:** 2025-11-11

**Authors:** Hongmin Jia, Yifan Li, Shanna Xu, Yuntao Xi, Weimin Gui

**Affiliations:** 1 School of Materials Science and Engineering, Xi’an Shiyou University, Xi’an 710065, China; 2 Shaanxi Fast Gear Co., Ltd., 129 West Avenue, Xi’an 710077, China

**Keywords:** Mg alloy, degradation behavior, mechanical integrity, immersion duration, localized corrosion

## Abstract

The mechanical deterioration of Mg alloys during degradation significantly impairs their service performance as biomaterial implants. In the present study, the degradation behavior of a Mg-6Zn-0.5Cu alloy was systematically examined through electrochemical measurements and immersion tests, while the mechanical integrity was assessed via tensile tests under different immersion periods. The results revealed a severe loss in mechanical properties was disproportionate to the corrosion rate. After 7 days’ immersion, the ultimate tensile strength (*UTS*) and elongation to failure (*EL*) decreased by 34.4% and 60.1%, respectively, while the corrosion rate was 0.11 mm/y based on the weight loss. This severe mechanical deterioration was primarily caused by pronounced localized corrosion, which induced aggravated local stress concentration at corrosion sites, promoting microcracks initiation and leading to premature fracture of the alloy.

## 1. Introduction

Mg and its alloys have emerged as promising implant materials due to their desirable mechanical properties, excellent biocompatibility, and remarkable biodegradability [1,2,3,4,5]. These intrinsic features make Mg alloys potential candidate to revolutionize orthopedic and cardiovascular implant applications, as they avoid the need for secondary removal surgeries, which is a common drawback of conventional metallic implants [1,6,7,8]. Nevertheless, the clinical application of Mg-based materials is significantly limited by a critical mismatch between the alloy corrosion and the tissue healing rate [9,10,11]. In physiological environment, the rapid degradation rate of Mg alloys often leads to premature loss of mechanical support, undermining the load-bearing capacity of biomedical implants.

Numerous in vivo and in vitro studies have been conducted to elucidate the degradation behavior of Mg alloys as implants [1,2,3,4,5,6,7,8,9,10,11,12,13]. However, most previous studies have focused either on the initial mechanical properties of Mg alloys or their corrosion rate [11,12,13,14,15,16,17]. Less emphasis has been placed on establishing the relationship between strength deterioration and degradation progression. It is crucial to recognize that the residual strength of Mg alloy throughout the degradation period plays a vital role in its service performance. Even with high initial strength, a Mg alloy holds little practical value if it fails to maintain mechanical stability throughout the required healing period. Furthermore, the connection between mechanical properties and degradation behavior in Mg alloys is not straightforward. Localized corrosion mechanisms, such as pitting corrosion, can markedly weaken mechanical integrity via stress concentration, even without a corresponding rise in mass loss or corrosion current density. This disparity highlights a serious clinical concern and reveals the limitations that solely focus on corrosion rate to assess the service reliability of Mg alloy implants.

Existing studies on the corrosion-related mechanical properties of Mg alloys mainly focus on corrosion fatigue and stress corrosion cracking [18,19,20,21], often exploring how external loads affect degradation. For Mg alloy implants, however, the primary concern lies in how physiologically corrosive environments influence the Mg matrix and its mechanical behavior, particularly the residual strength changes over time. Despite its critical importance for ensuring the safety and efficacy of Mg alloy implants, research quantitatively linking degradation with the loss of load bearing capacity remains relatively scarce. This gap poses a significant challenge in predicting the mechanical integrity safety window for biodegradable implants, which is crucial for avoiding premature in vivo failures.

The present study focuses on investigating the effects of immersion durations in Hank’s solution on the degradation rate and mechanical deterioration of a Mg-6Zn-0.5Cu alloy. Through a combination of corrosion measurements and tensile tests, this work seeks to establish the correlation between degradation progression and decline in mechanical integrity. The findings are expected to provide a possible framework for estimating the residual strength of Mg alloy, thereby supporting the design and development of safer Mg alloy implants with more predictable service performance.

## 2. Materials and Methods

### 2.1. Sample Preparation

The Mg-6Zn-0.5Cu alloy was produced via conventional casting method using a resistance furnace. High purity Mg, Zn and Cu (Shanxi Yunguang Huasheng Magnesium Co., Ltd., Yuncheng, China) were melted under a protective atmosphere of CO_2_ and SF_6_, and the molten metal was poured into a mold preheated to 200 °C. Following casting, the ingot was homogenized at 380 °C for 24 h, and was subsequently extruded at 340 °C with an extrusion ratio of 39:1 and a constant ram speed of 0.7 m/min. The chemical composition of the alloy was determined by inductively coupled plasma (ICP) spectrometry (IRIS Intrepid, Trenton, NJ, USA), as detailed in Table 1. For microstructural examination and degradation analysis, specimens with dimensions of 10 mm × 10 mm × 5 mm were cut from the extruded bar along extrusion direction using wire electrical discharge machining.

### 2.2. Microstructure Characterization

The microstructural feature of the samples was examined using an optical microscope (OM, ZEISS Axio Observe. Z1m, Jena, Germany), scanning electron microscopy (SEM, JSM-6460, Tokyo, Japan), and electron backscatter diffraction (EBSD, Abingdon, UK). Samples for OM and SEM were ground, polished and etched in 4% nitrate alcohol solution. EBSD measurement was performed with an HKL Channel 5 system with a step size of 0.5 µm. Phase identification was conducted by X-ray diffraction (XRD, Philips PW170X, Almelo, The Netherlands) with Cu Kα radiation over a 2θ range of 20° to 90°.

### 2.3. Degradation Behavior

The degradation behavior of the alloy was investigated through electrochemical tests and immersion measurements in Hank’s solution for durations of 1, 3, 5 and 7 days. A non-pre-immersed sample served as the control, and the composition of Hank’s solution is detailed in Table 2.

A conventional three-electrode system in a 500 mL electrolytic cell was employed for electrochemical measurements (DH 7001, Wuhan CorrTest Instruments Co., Ltd., Wuhan, China). The working electrode was the sample with an exposed surface area of 1 cm^2^ embedded in epoxy resin, while a platinum sheet and an Ag|AgCl electrode were used as the counter and reference electrodes, respectively. Prior to testing, the sample surface was sequentially ground using SiC abrasive papers up to 2000 grit, and then ultrasonic cleaned in deionized water and ethanol. The electrochemical evaluation involved monitoring the open circuit potential (OCP), along with conducting potentiodynamic polarization and electrochemical impedance spectroscopy (EIS). The OCP was tracked for 900 s to confirm system stability. Polarization scans were performed from −400 mV to 400 mV (vs. RE) at a scanning rate of 0.1667 mV/s. EIS was measured across a frequency spectrum of 100 kHz to 10 mHz, applying an AC perturbation amplitude of 10 mV (vs. RE). All electrochemical tests were carried out at least three samples to ensure reproducibility.

To determine the corrosion rate, weight loss was measured after periods of 1, 3, 5 and 7 days. Following immersion, the corrosion products were chemically removed in a 20% CrO_3_ solution at 80 °C for 5 min. After drying, the specimens were reweighed. The surface morphologies after different immersion periods were then characterized by SEM using an accelerating voltage of 30 kV. Each experiment was repeated at least three times to ensure accuracy.

### 2.4. Mechanical Integrity After Degradation

**Figure 1:**
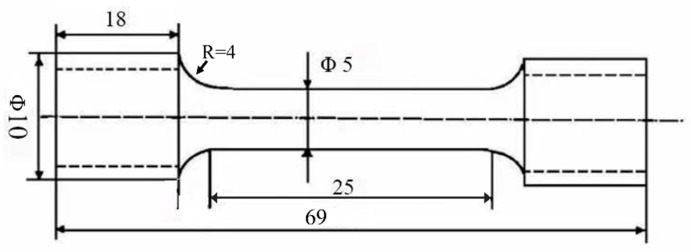
Detail dimension of the tensile testing specimen.

Tensile tests were performed on an AG-100kNG universal testing machine (Shimadzu Co. Ltd., Tokyo, Japan) with a crosshead speed maintained at 1 mm·min ^−1^. Dog-bone-shaped samples were machined following the GBT 228.1-2021 standard [22], the detailed dimensions were provided in Figure 1. To evaluate the mechanical degradation, specimens were immersed in Hank’s solution for 1, 3, 5 and 7 days (denoted as 1 d, 3 d, 5 d, and 7 d in the figures, respectively) before testing, using a non-pre-immersed sample as the control. During immersion, the clamping sections of the specimens were sealed with silicone rubber to avoid corrosion. After tensile failure, the fractured surface was examined using SEM. Each testing condition was replicated at least three times to ensure reproducibility.

## 3. Results

### 3.1. Microstructure

**Figure 2:**
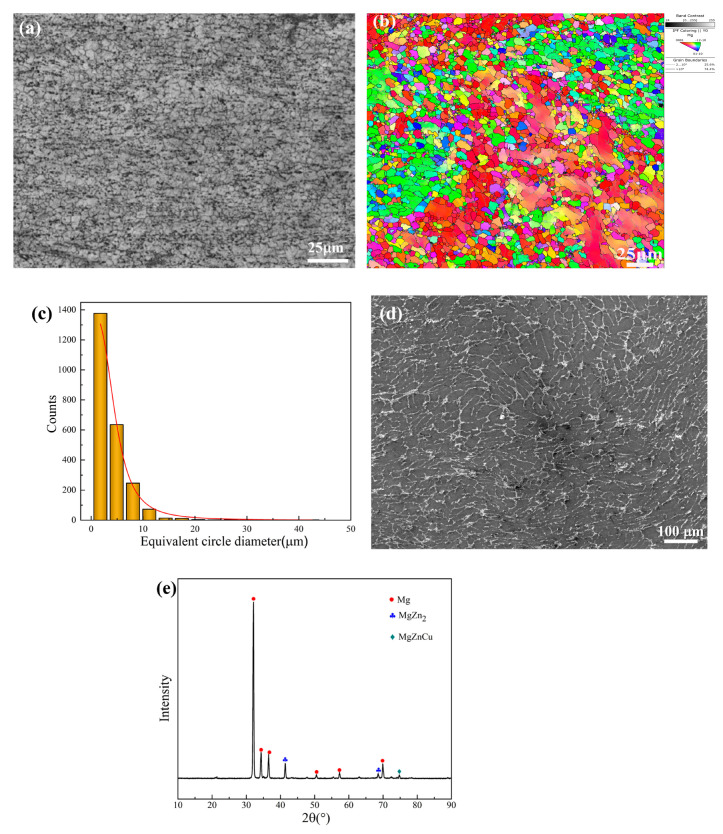
Microstructure of as-extruded Mg-6Zn-0.5Cu alloy characterized by (**a**) OM and (**b**) EBSD, (**c**) the distribution of grain size, (**d**) SEM image and (**e**) XRD pattern.

The microstructure of the as-extruded Mg-6Zn-0.5Cu alloy, examined by OM and EBSD, is displayed in Figure 2a,b. The alloy consists mainly of equiaxial grains with an averaged grain size of 3.95 µm, indicating that complete dynamical recrystallization occurred during the hot extrusion process. As revealed by the SEM image in Figure 2d, the secondary phases are distributed as a continuous network along the grain boundaries. Phase identification by XRD (Figure 2e) confirms that these secondary phases are MgZn_2_ and MgZnCu.

### 3.2. Degradation Characteristics of Mg-6Zn-0.5Cu Alloy Under Varying Immersion Durations

(1) Electrochemical behavior

**Figure 3:**
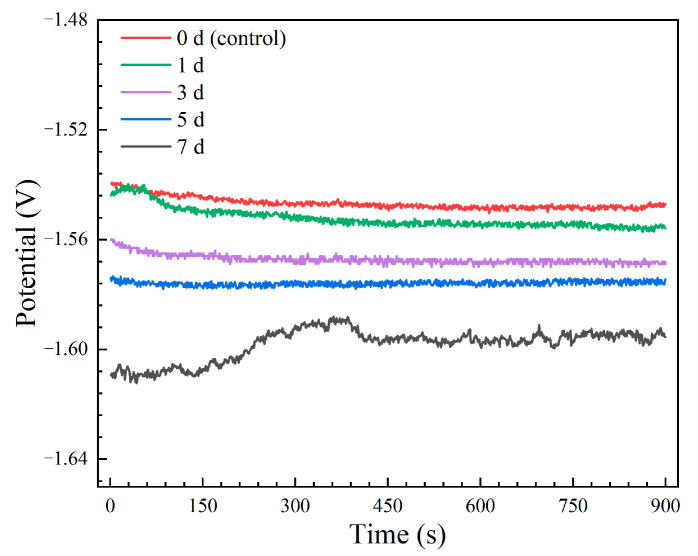
OCP curves of the Mg-6Zn-0.5Cu alloy under varying immersion durations in Hank’s solution.

The open circuit potential (OCP) curves of the Mg-6Zn-0.5Cu alloy after different immersion durations are presented in Figure 3. All curves exhibit relatively stable potentials with minor fluctuations, which can be attributed to the dynamic formation and detachment of corrosion products [23,24]. The electrochemical activity, ranked as 7 days > 5 days > 3 days > 1 day > 0 day (control), increases with immersion duration. The sample immersed for 7 days exhibits the most negative OCP value, indicating the highest susceptibility to corrosion. While the non-pre-immersed control, with the noblest potential, demonstrates the lowest tendency. This trend suggests that prolonged immersion duration enhances the electrochemical activity of the alloy, which may be due to changes in its surface state.

**Figure 4:**
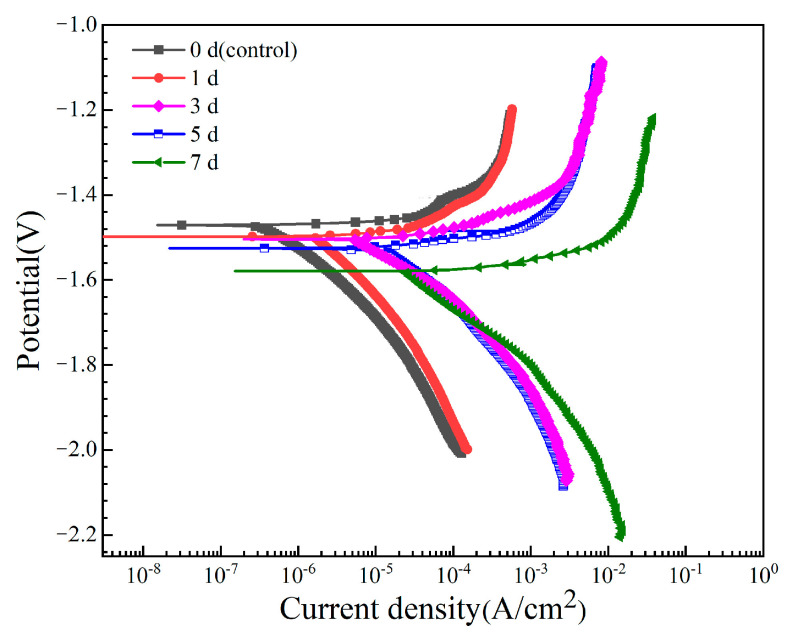
Potentiodynamic polarization curves of the Mg-6Zn-0.5Cu alloy under varying immersion durations in Hank’s solution.

The potentiodynamic polarization curves of the Mg-6Zn-0.5Cu alloy after varying immersion time in Hank’s solution are shown in Figure 4. Key electrochemical parameters derived by Tafel extrapolation, including corrosion potential (*E_corr_*) and corrosion current density (*i_corr_*), are listed in Table 3. The results indicate a clear trend: *E_corr_* shifts negatively and *i_corr_* increases with prolonged immersion. In general, a lower *i_corr_* value implies higher corrosion resistance. Notably, the *i_corr_* value after 7 days’ immersion is two orders of magnitude higher than that of the non-pre-immersed sample, revealing enhanced electrochemical activity and the inability of the corrosion layer to protect the substrate.

**Figure 5:**
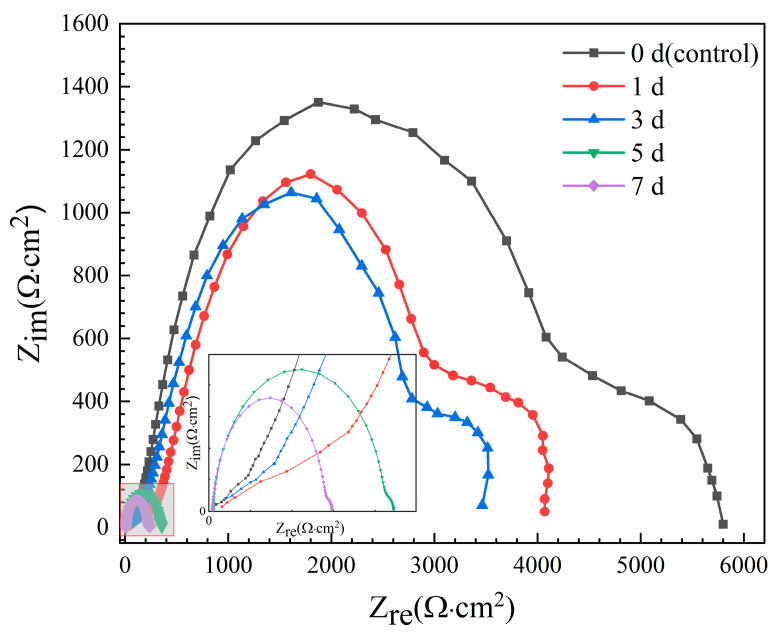
Nyquist spectra of the Mg-6Zn-0.5Cu alloy under varying immersion durations in Hank’s solution.

EIS Nyquist spectra of the Mg-6Zn-0.5Cu alloy after different immersion durations are presented in Figure 5. The spectra consistently feature two capacitive loops in high (10^1^–10^4^ Hz) and medium frequency (10^−2^–10^1^ Hz) regions, representing the surface film resistance (*R*_f_) and the charge transfer resistance (*R*_ct_) related to the corrosion reaction kinetics [25,26,27,28,29]. Since a larger loop radius signifies greater corrosion resistance, the observed sharp contraction in loop diameters with longer immersion duration indicates a rapid reduction in the corrosion resistance of the samples. This conclusion is consistent with the polarization results in Figure 4, which shows that the increased immersion time leads to higher corrosion current density and faster degradation rate.

(2) Weight loss and corrosion morphologies

**Figure 6:**
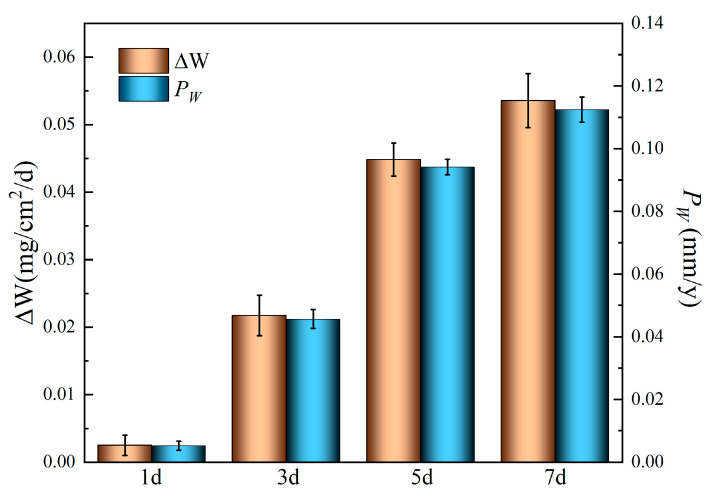
Weight loss and degradation rates of the Mg-6Zn-0.5Cu alloy in Hank’s solution for different immersion time.

Figure 6 presents the weight loss (*ΔW*, mg∙cm^−2^∙d) and corresponding degradation rates (*P_w_*, mm/y) of the alloy immersed in Hank’s solution over different immersion periods, where *P_w_* is derived from *ΔW* using Equation (1) [25,26,27]. The *ΔW* value starts at approximately 0.0025 mg∙cm^−2^∙d after 1 day and increases to 0.022 mg∙cm^−2^∙d after 3 days, and the *P_w_* increases from 0.005 mm/y to 0.046 mm/y over the same interval. After 7 days’ immersion, the degradation rate reaches 0.11 mm/y, representing a value 22 times higher than the initial rate. These results indicate a progressive acceleration of degradation process with the prolonged immersion, consistent with the electrochemical measurement.

*P_w_* = 2.10 *ΔW* (1)

**Figure 7:**
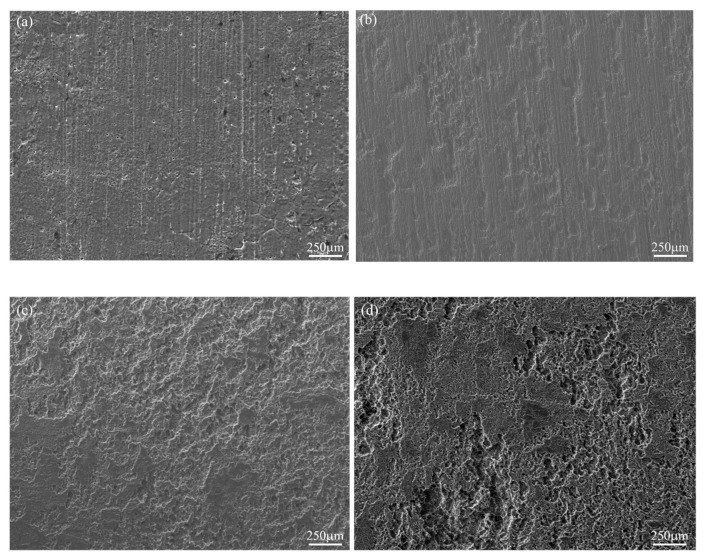
Corrosion morphologies of the Mg-6Zn-0.5Cu alloy under different immersion durations in Hank’s solution, characterized by SEM at 50× magnification. (**a**) 1 day, (**b**) 3 days, (**c**) 5 days and (**d**) 7 days, respectively.

The corrosion morphology evolution of the Mg-6Zn-0.5Cu alloy in Hank’s solution for varying immersion durations was characterized using SEM at 50× magnification, as shown in Figure 7. The results demonstrate that the alloy suffers from localized corrosion. After 1 day’s immersion, only a limited number of shallow, small pits are visible on the surface. With the immersion time extended to 3 days, the corroded area expands and the pit dimensions increase noticeably. After 5 days of immersion, uniform corrosion damage covers the entire surface, accompanied by the appearance of deep and severe pits. Further exposure leads to increasingly aggravated corrosion features, as shown in Figure 7d. This morphological evolution aligns with the electrochemical results in Figure 4 and Figure 5, confirming the increasing susceptibility to corrosion over exposure time.

### 3.3. Mechanical Integrity of Mg-6Zn-0.5Cu Alloy After Varying Immersion Durations

**Figure 8:**
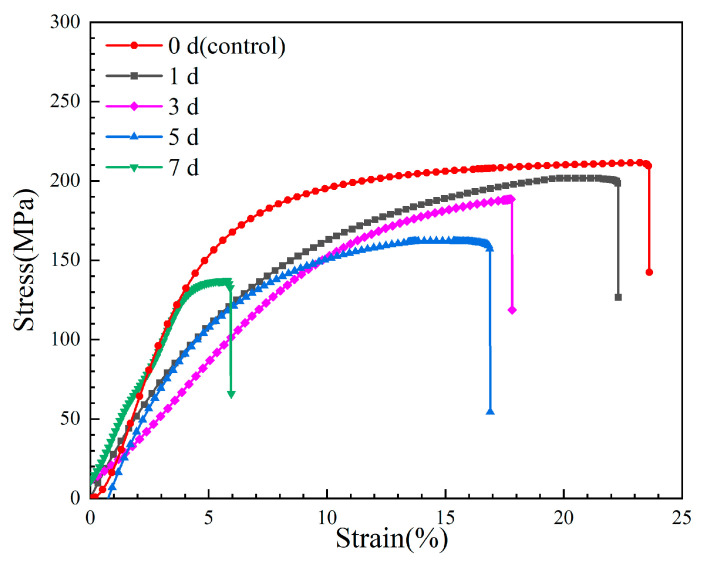
Stress–strain curves of the Mg-6Zn-0.5Cu alloy under varying immersion durations in Hank’s solution.

**Figure 9:**
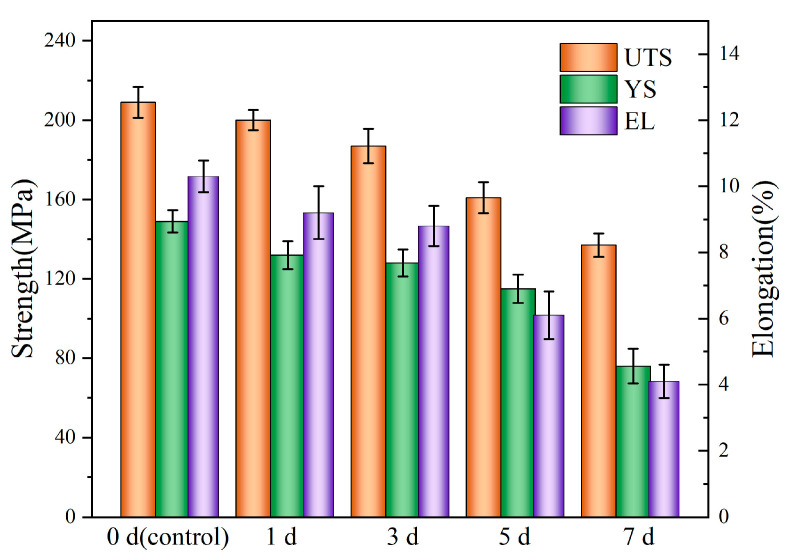
Strength and elongation of the Mg-6Zn-0.5Cu alloy under varying immersion durations in Hank’s solution.

Figure 8 shows the stress–strain curves of the Mg-6Zn-0.5Cu alloy after varying immersion durations, and the red curve represents the original mechanical properties of the tested alloy. The corresponding mechanical properties of the alloy after 1, 3, 5 and 7 days immersion are plotted in Figure 9. For the non-pre-immersion sample, the ultimate tensile strength (*UTS*), yield strength (*YS*) and elongation (*EL*) are 209 MPa, 149 MPa and 10.3%, respectively. As the immersion time increases, the *UTS*, *YS* and *EL* of the alloy progressively decrease. After 7 days’ immersion in Hank’s solution, the *UTS*, *YS* and *EL* of the alloy decrease to 137 MPa, 76 MPa and 4.1%, corresponding to reductions of 34.4%, 48.9% and 60.1%, respectively, compared to the non-pre-immersed alloy.

**Figure 10:**
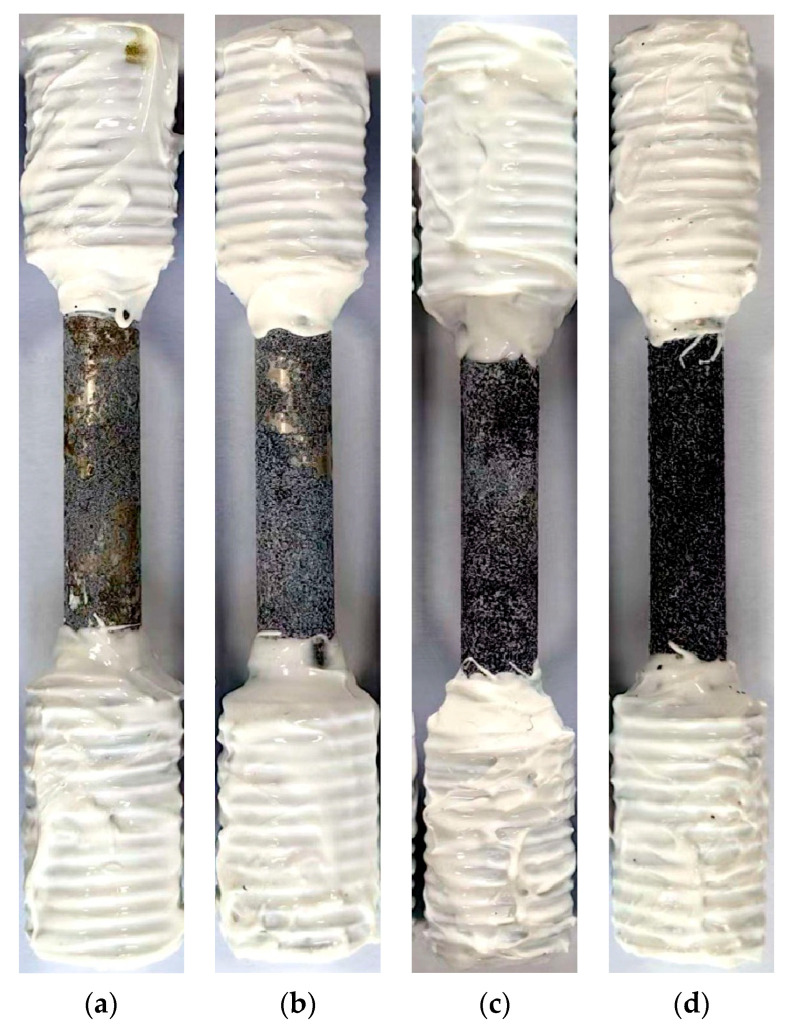
Macroscopic surface morphologies of the Mg-6Zn-0.5Cu alloy under varying immersion durations in Hank’s solution. (**a**) 1 day, (**b**) 3 days, (**c**) 5 days and (**d**) 7 days, respectively.

Figure 10 displays the macroscopic surface morphologies of the Mg-6Zn-0.5Cu alloy after being immersed in Hank’s solution for 1, 3, 5 and 7 days. All surfaces show accumulation of corrosion products, the extent of which depends on the immersion time. Slight deposition after 1 day of exposure evolves into heavy coverage with prolonged exposure, reflecting progressively more severe corrosion damage.

**Figure 11:**
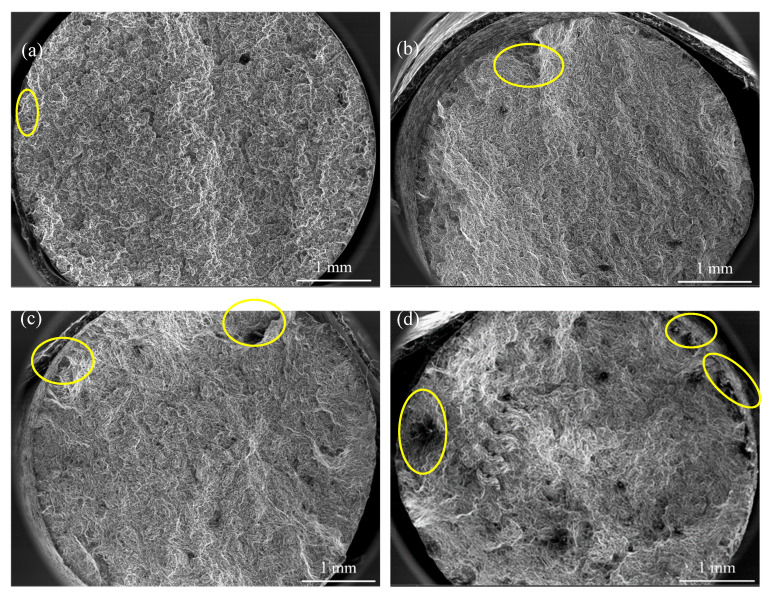
Fracture surfaces of the Mg-6Zn-0.5Cu alloy under varying immersion durations in Hank’s solution, characterized by SEM at 37× magnification. (**a**) 1 day, (**b**) 3 days, (**c**) 5 days and (**d**) 7 days, respectively.

The fracture surfaces of tensile samples were examined by SEM at 37× magnification to assess the influence of immersion time on the mechanical integrity, which are shown in Figure 11. The alloy displays a mixed fracture mode across different immersion times, characterized by quasi-cleavage features. Localized corrosion pits of varying severities were observed along the edges of the tensile bars and spread radially. The density of these corrosion pits increased with the exposure time. During tensile loading, such corrosion pits act as stress concentrators, initiating crack formation and propagation. Although the fracture mechanism remains unchanged, the presence of localized corrosion pits significantly undermines the mechanical integrity of the alloy.

## 4. Discussion

Microstructural and phase analyses presented in Figure 2 reveal that the secondary phases, primarily MgZn_2_ and MgZnCu, are predominantly distributed in a continuous network along grain boundaries. These phases play a decisive role in the alloy’s degradation mode. Due to the high intrinsic reactivity of Mg (−2.36 V_SCE_), these secondary phases act as the cathodic sites relative to the anodic α-Mg matrix. Volta potential measurements reveal that the MgZn_2_ and MgZnCu phases exhibit nobler potentials, approximately 320 mV and 680 mV higher than the α-Mg matrix, respectively [30]. This pronounced electrochemical disparity, combined with the continuous distribution of these cathodic phases, promotes extensive galvanic coupling throughout the alloy. Consequently, the α-Mg matrix is preferentially corroded, initiating severe micro-galvanic attack that propagates aggressively along grain boundaries. The corresponding corrosion reactions can be described as Equations (2) and (3) [19,27,28].

Mg → Mg + 2e^−^ (2)

2H_2_O + 2e^−^ → 2OH^−^ + 2H_2_ (3)

This microstructure-driven corrosion behavior dictates both the degradation rate and the initiation of localized pits that significantly impair mechanical integrity. To quantitively correlate the degradation rate and mechanical integrity for the tested alloy, the evaluation parameters *Δ_UTS_*, *Δ_YS_*, *Δ_EL_* (reduction rates of ultimate tensile stress, yield stress and elongation, respectively) [1] are defined as follows:

(4) $$\Delta_{UTS}\;=\;\frac{{UTS}_{initial}-{UTS}_{x}}{{UTS}_{initial}}\;\times \;100\%$$

(5) $$\Delta_{YS}=\frac{{YS}_{initial}-{YS}_{x}}{{YS}_{initial}}\times 100\%$$

(6) $$\Delta_{EL}=\frac{{EL}_{initial}-{EL}_{x}}{{EL}_{initial}}\times 100\%$$

Here, *x* represents the immersion duration, and the subscript initial refers to non-pre-immersed control group.

**Figure 12:**
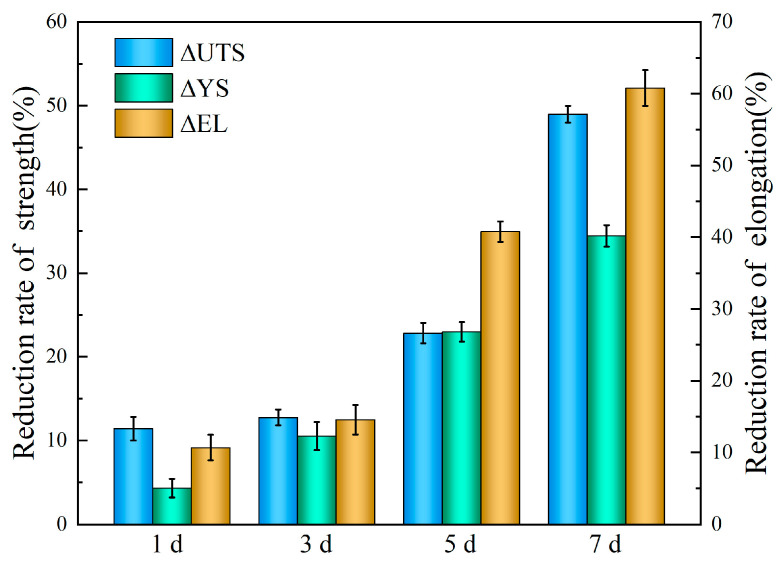
Reduction rate of mechanical properties of the Mg-6Zn-0.5Cu alloy in Hank’s solution under varying immersion durations.

As shown in Figure 12, the trends in $\Delta_{UTS}$, $\Delta_{YS}$, and $\Delta_{EL}$ reveal that the immersion duration exhibits a remarkable influence on mechanical integrity. After 7 days’ immersion, the *UTS*, *YS*, and *EL* decreased by 34.4%, 48.9% and 60.1%, respectively. Based on weight loss measurements and calculation (Figure 6), the degradation rate of samples immersed for 7 days is 0.11 mm/y. Although this result represents a marked acceleration in degradation, it still meets the requirement for biomedical materials [31], which require a corrosion rate below 0.5 mm/y for medical implant materials. These results indicate that immersion time affects mechanical integrity more profoundly than it does the degradation rate.

A positive correlation is further identified between degradation rate and the loss of mechanical properties. In Hank’s solution, localized corrosion of Mg alloy creates an alkaline environment, as described in Equations (2) and (3). This environment promotes the formation of protective corrosion products by interactions among Mg^2+^, Ca^2+^, HCO_3_^−^, and HPO_4_^2−^ from the solution, partially decelerating the overall degradation rate. Nevertheless, localized corrosion remains the dominant mechanism, and the high concentration of Cl^−^ in the solution further encourages uniform corrosion [32,33], leading to the development of corrosion pits, as evidenced in Figure 7 and Figure 11. Surface corrosion pits serve as a primary factor affecting their mechanical properties. Under tensile loading, these pits act as local stress concentration sites, initiating microcracks in adjacent regions. The loss of effective load bearing cross-sectional area due to localized corrosion reduces the sample’s overall capacity to sustain stress. Under sustained tensile stress, localized stress intensifies significantly, promoting the growth of these microcracks and ultimately leading to fracture. As immersion time increases, the increasing density of corrosion pits results in aggravated local stress concentration. Consequently, the mechanical properties of the alloy deteriorate significantly after 7 days’ immersion.

## 5. Conclusions

This study systematically investigated the degradation behavior and mechanical integrity evolution of a Mg-6Zn-0.5Cu alloy in Hank’s solution under varying immersion periods. The main conclusions are as follows:

(1) After 7 days’ immersion, the *UTS* and *EL* decreased markedly by 34.4% and 60.1%, respectively. While the weight loss of 0.054 mg∙cm^−2^∙d corresponds to a degradation rate of 0.11 mm/y.
(2) The results indicate that the alloy is susceptible to localized corrosion under this physiological environment with mechanical deterioration occurring at a rate that exceeds the corrosion.
(3) The pronounced loss in mechanical performance is attributed to localized corrosion associated with secondary phases. Under tensile testing, the resulting corrosion pits serve as stress concentrators, promoting microcrack initiation and leading to premature fracture of the alloy.
(4) Even a modest corrosion rate can result in severe mechanical degradation due to localized attack, highlighting the limitation of relying solely on corrosion rate to predict the service life of biodegradable Mg implants.

## Figures and Tables

**Table 1:** The chemical composition of Mg-6Zn-0.5Cu alloy measured by ICP (wt.%).

Element	Zn	Cu	Mg
Content	6	0.5	Bal.

**Table 2:** Chemical composition of Hank’s solution (g/L).

NaCl	KCl	CaCl_2_	Na_2_HPO_4_∙7H_2_O	MgSO_4_∙7H_2_O	NaHCO_3_	KH_2_PO_4_	C_6_H_12_O_6_
8	0.4	0.14	0.09	0.2	0.35	0.06	1

**Table 3:** The electrochemical parameters of the Mg-6Zn-0.5Cu alloy after varying immersion time in Hank’s solution.

Time (d)	*E_corr_* (V)	*i_corr_* (A/cm^2^)
0	−1.482 ± 0.016	(6.28 ± 0.153) × 10^−7^
1	−1.501 ± 0.018	(2.05 ± 0.129) × 10^−6^
3	−1.507 ± 0.019	(6.19 ± 0.190) × 10^−6^
5	−1.528 ± 0.027	(1.71 ± 0.341) × 10^−5^
7	−1.591 ± 0.044	(3.06 ± 0.553) × 10^−5^

## Data Availability

The original contributions presented in this study are included in the article material. Further inquiries can be directed to the corresponding authors.
